# Thermostat wars? The roles of gender and thermal comfort negotiations in household energy use behavior

**DOI:** 10.1371/journal.pone.0224198

**Published:** 2019-11-13

**Authors:** Nicole D. Sintov, Lee V. White, Hugh Walpole

**Affiliations:** School of Environment and Natural Resources, The Ohio State University, Columbus, Ohio, United States of America; University of Maryland College Park, UNITED STATES

## Abstract

Although advanced thermostat technologies offer energy efficiency potential, these devices alone do not guarantee savings. Household occupants often deviate from thermostat programs, perhaps due to differing thermal comfort preferences, which are strong drivers of residential energy use and vary across genders. This study aims to develop an initial typology of interpersonal interactions around thermal comfort, explore the role of gender in such interactions, and examine the impacts of interactions on thermostat adjustments. Using n = 1568 diary observations collected from 112 participants, we identify three interaction types: conflicts, compromises, and agreements. Fixed effects analyses find that women are marginally more likely to report engaging in conflicts, whereas men are significantly more likely to report engaging in agreements and compromises, both of which are associated with greater likelihood of adjusting thermostats within a given day. This work represents an early step in investigating the multiply determined nature of household energy decisions.

## Introduction

Heating and cooling combined comprised 32% of U.S. residential energy consumption in 2015 [1], while available data from 2010 place that share around 41% worldwide [2]. Targeting residential thermal management systems and behaviors therefore offers considerable potential for reducing home energy use. To this end, advances in programmable and smart thermostat technologies offer both convenience as well as potential energy efficiency gains [3]. For instance, prior work estimates that employing the thermostat set-backs available on programmable thermostats can save up to 14% of home energy used for heating [4] and up to 17% used for cooling [5]. However, people do not always follow the programs that they set on such devices, if they set such programs in the first place [6,7]. This can lead to households with programmable thermostats (i.e., those that can be set to automatically adjust the temperature at particular times of day) or smart thermostats (internet enabled thermostats that can be adjusted using a smart phone or other internet-enabled device, and/or that can “learn” occupant preferences) not exploiting the full efficiency potential of these technologies [6,8–10]. In fact, ENERGY STAR ceased labelling programmable thermostats as energy saving devices in 2009, citing unreliable energy savings given that savings are not solely dependent on thermostat, but also on occupant behavior [10].

## Variation in thermal comfort preferences across occupants

These circumstances underline the need to identify factors that contribute to occupants manually adjusting programmable thermostats rather than allowing them to run their programs. Previous research has identified a variety of thermostat design aspects that can influence the ease and confidence with which people program thermostats [8,9]. However, deviations from thermostat programs are likely not due entirely to a lack of knowledge or design flaws; rather, they may also be due to differing thermal comfort preferences between household occupants [11,12]. According to the adaptive model of thermal comfort, thermal comfort arises from three primary factors: physiological acclimatization, behavioural adjustment (e.g., adding or removing clothing layers), and psychological expectations (perceptions of sensory inputs influenced by past experiences and expectations), with the first factor being less important relative to the latter two [13–15]. In particular, work on psychological adaptation finds perceived control over temperature to be amongst the strongest predictors of thermal comfort ratings [16]. Further, thermal (dis)comfort is dynamic–it can evolve over time (e.g., ‘temporal alliesthesia’) [17]. Given the complexity in this set of thermal comfort determinants, it is not surprising that there is considerable inter-individual variation in thermal comfort preferences [11,18]. In particular, focusing on gender differences, a rich body of work has found that women tend to prefer warmer environments, be satisfied with a narrower band of thermal environments, and feel uncomfortable more often than men; they also perform better on cognitive tasks in warmer environments than do men, and vice versa [11,19–21]. Prior work has found that differences in typical clothing worn by women vs. men, as well as physiological and metabolic differences, can contribute to these findings [21]. There may also be gender differences in psychological expectations pertaining to thermal comfort.

Thermal comfort preferences are strong negative predictors of energy conservation intentions and behavior [19,22], and as mentioned above, efforts to maintain thermal comfort typically consume a considerable proportion of energy used in homes [23]. Given that the vast majority of residences include more than one adult occupant [24], unique (often differing) preferences around thermal comfort may well influence thermostat adjustments and energy use, as occupants attempt to negotiate their varying preferences. Such inter-individual differences are a key reason that other researchers advocate for variable (vs. uniform) standards for indoor temperatures in commercial spaces [13,14,25], and for giving occupants individual-level control over their thermal environments [15,17]. Identifying a temperature set-point agreeable to all home occupants remains a considerable challenge that has been highlighted in prior work [26].

### The overlooked role of interpersonal interactions in household energy use

Similar to other household-level decisions (e.g., purchasing groceries, appliances, internet service), choices about managing the household thermostat likely diverge from the preferences of any individual occupant, and rather arise from the preferences of multiple occupants. Of critical importance is how occupants negotiate their differing preferences and arrive at a decision. Although emerging research suggests that discussing energy use is linked with energy efficiency behaviors [27,28], and a few qualitative studies have noted conflicts between genders over thermostat settings [29], to date, no research to the authors’ knowledge has directly examined how interactions between household occupants may contribute to thermostat use or other energy use behaviors. This gap may exist be in part because most research on household energy consumption behavior relies on self-reports from a single occupant (an individual-level approach), whereas energy consumption is typically measured at the household level (e.g. household kWh usage or bills [30,31]). These approaches entail an inherent mismatch between the levels of psychological (a single occupant’s preferences, decisions, and/or behaviors) and physical (multiple household occupants contributing to household-level energy use) measurement. Researchers, including authors of the present study, often make the implicit assumption that household-level measures of energy use accurately represent the preferences and behaviors of individual occupants within the home. However, such an assumption fails to account for variation in preferences across occupants, as well as the interactions among them.

### Gender and (thermal comfort) negotiations

Prior work finds that individual differences (e.g., demographic characteristics) can influence the likelihood of engaging in certain types of conversations (e.g., about science) [32]. As many households include occupants of different genders, it is necessary to consider the potential role of gender in household interactions around thermal comfort. Drawing from research on negotiation, a large body of work has found that men tend to achieve better outcomes than women in economic negotiations [33]. Additionally, women are less likely than men to initiate a negotiation in the first place; rather, women are more likely to engage when the opportunity to do so is framed politely as “asking” rather than negotiating, the latter of which is perceived by women (but not men) as more intimidating [34]. A host of both situational and personal factors that can be understood as byproducts of gender socialization help explain the difference in women’s vs. men’s likelihood of engaging in and outcomes of negotiation [35]. Social role theory suggests that gender roles consist of beliefs about expectations and norms related to men’s and women’s roles [36]. Whereas the woman gender role has characteristics such as being accommodating and relationship-oriented [36], the man gender role has agentic characteristics such as behaving in competitive or assertive ways. Further, role congruity theory [37] suggests that gender differences in negotiation behavior and outcomes can be explained by the fact that the agentic behaviors usually considered key in negotiating tend to be incongruent with the woman gender role. In other words, women who engage in agentic behaviors (i.e., incongruent with the woman gender role) may risk social rejection. Further, negotiations are dynamic, marked by events and processes that can evolve over the course of a negotiation. Disagreements may arise, necessitating consideration of how such impasses may be addressed. A meta-analysis of conflict resolution styles in workplace settings found that relative to men, women in individualistic cultures are more likely to endorse compromising and less likely to use strategies that elevate their own or production goals while sacrificing the desires of others (e.g., forcing) [38], also consistent with gender roles.

Overall, the literature suggests that women are less likely to negotiate for their needs in the first place, and when engaged in a negotiation, may be less likely to achieve desired outcomes, particularly if there is a disagreement, in which case they may compromise or defer to others (relationship orientation). As much of the negotiation literature focuses on economic negotiations, it is unclear if the findings on gender differences translate to other contexts, such as the household. In particular, thermal comfort and economic negotiations differ because the outcome of adjusting the home thermostat has implications for all involved parties (i.e., comfort), vs. primarily the negotiator (i.e., salary). In such a situation, woman gender role- congruent actions would focus on considering others’ needs, so women may be less likely than men to initiate thermal comfort discussions to begin with, and may defer to others’ preferences when such an interaction occurs. On the other hand, higher levels of familiarity and comfort characterizing domestic relationships might reduce women’s perceived risk of social rejection arising from gender role incongruence, possibly elevating her likelihood of initiating a thermal comfort negotiation. Also, prior work finds that when gender stereotypes are explicitly activated (in this case, “women are always cold”), this may lead to reactance, whereby women embrace man gender-role congruent traits (e.g., assertiveness) [39]. This could lead to women advocating more strongly for their needs in a negotiation, and possibly to conflict, if such gender-incongruent assertiveness is perceived negatively by women’s negotiation partners.

### Present study objectives, research questions, and hypotheses

The primary goals of the present study are to develop an initial typology of interactions that arise around thermal comfort, explore the role of gender in such interactions, and examine the impacts of these interactions on thermostat adjustments. We address four research questions. First, to replicate previous work and provide a basis for subsequent hypotheses, we examine gender differences in subjective and experienced thermal comfort. We hypothesize that women will prefer warmer (H1a) and men cooler (H1b) thermal environments, and that women will experience thermal discomfort more frequently than men (H2). Third, we address whether occupants’ genders and experiences of thermal discomfort influence types of interpersonal interactions that occur. We hypothesize that when household occupants experience thermal discomfort on a given day, interactions of all types will be more likely to occur on that day (H3a), that women will be more likely to report conflict (H3b), and men will be more likely to report agreement (H3c). Finally, we investigate whether the type of interaction occupants have on a given day influences the likelihood of making a thermostat adjustment on that day. We predict that agreements will be associated with fewer adjustments (H4a), and that conflicts will be associated with more adjustments (H4b).

### Contributions

This study makes several contributions. It is the first to the authors’ knowledge to examine types of interactions around thermal comfort, and evaluate their influence on energy use behavior. Additionally, this work extends social role theory by examining gender differences in discussions of thermal comfort, a unique behavioral context wherein we could reasonably expect the circumstances to encourage women to either act in accordance with or react against role-congruent norms.

## Methods

Participants in this study completed a baseline survey and responded to a daily diary for two weeks. They were compensated up to $10 (distributed in two $5 increments) in e-gift card links. OSU’s Institutional Review Board (IRB) reviewed all study procedures and deemed the study exempt.

### Procedures

#### Recruitment

In Autumn 2017, trained research assistants conducted door-to-door recruitment, primarily on weekday afternoons and evenings. We targeted zip codes in Columbus, Ohio, with relatively high concentrations of multi-occupant households based on census data. To avoid revisiting homes, research assistants began recruiting along the outer edge of each zip code and worked their way to the center.

To be eligible for inclusion in the study, participants had to be at least 18 years of age, reside in a dwelling with an adjustable thermostat with at least one other person, pay their own electric bill, and own a mobile phone. Additionally, participants had to provide both survey and diary data. Participants in this study are individuals reporting on behalf of their households; initial attempts to recruit dyads were not successful. Eligibility was determined at the door via a series of verbal questions.

#### Remind

Remind is a cloud-based service that allows real-time, two-way communication via the Remind mobile application (app), text message, or email. The research team used Remind to enroll participants in the study, distribute online survey URLs, send reminders and daily diary prompts, deliver incentives, and receive participants’ diary reports, all while maintaining participant anonymity. To enroll, participants entered a username of their choice, along with a phone number or email, into the app on the research assistant’s phone, thus adding themselves to the study’s “group”. Participants could opt to receive communications and respond back to the study team (e.g., to diary prompts) via the Remind app (participants could download to their mobile phones if desired), text, and/or email.

#### Survey

Still at the door, research assistants sent participants a survey URL via Remind. The survey took approximately 5–10 minutes, and could be completed at any time on any internet-connected device. Upon survey completion, participants received the first $5 incentive.

#### Diaries

After completing the survey, participants were moved to the diary phase of the study, during which the research team used Remind to send participants the same two prompts each night for two weeks. As mentioned above, participants could respond to diary prompts via the Remind app, email, or text.

### Materials

#### Recruitment

During recruitment, the primary materials used by research assistants included smartphones with internet access and the Remind app.

#### Survey measures and coding

The survey assessed information about occupants’ thermal comfort preferences, home thermostats, energy bill consciousness, household size and composition, and demographic characteristics. Details for key measures can be found in S1 Appendix.

Participants’ subjective thermal comfort preferences were assessed using established scales [22] that indicate subjective preferences for cool and warm thermal environments. Two items assessed preference for warm environment (e.g., “While others might tolerate lowering their thermostat settings in the winter, my own need for being warm is high”), and three items assessed preference for a cool environment (e.g., “While others might turn off their air conditioners in the summer, my own need for being cool is high”). Items were rated on a Likert scale ranging from 1 = strongly disagree to 7 = strongly agree. The means of the two warmth and three cool items were taken to form scale variables indicating preference for warmth (Cronbach’s α = .76), and cool (Cronbach’s α = .83), respectively. Energy bill consciousness was assessed using three items [22] (e.g., “I keep track of my monthly electricity bills”), each of which was rated on a Likert scale ranging from 1 = strongly disagree to 7 = strongly agree. The mean of the three items was taken to form a scale variable indicating energy bill consciousness (Cronbach’s α = .73). The survey also assessed whether or not the home had a programmable thermostat, and whether or not the thermostat was programmed (1 = yes, 0 = no).

Demographics included participants’ gender (0 = man, 1 = woman, 2 = other), age (continuous), educational attainment (Did not complete high school, High school/GED, Some college/associates degree, 4-year college degree, Graduate degree), ethnicity (of Hispanic or Spanish origin, not of Hispanic or Spanish origin), race (White or Caucasian, Black or African American, American Indian, or Aleut, Asian or Pacific Islander, Multiracial, Other), and political orientation (rated on a Likert scale ranging from 1 = extremely liberal to 7 = extremely conservative). Also assessed were household income (1 = <USD$15,000 to 9 = >USD$200,000), and homeownership status. Household composition was assessed by asking about the number of other occupants in the home, and whether occupants were over or under the age of 18.

#### Diary data coding and measures

Participants were asked to respond to two diary prompts each night for two weeks, as follows: (1) “Did you or anyone else in your household adjust the thermostat in your home today? What adjustments were made and by whom?” and (2) “Others in your home may have different thoughts about how warm or cool it is in the house. Tell us about any related discussions you had”. Participants’ responses to each question were collected through the Remind service daily for 14 days. An iterative coding process was used on the resulting dataset [40]. Specifically, two trained coders independently coded each response for a set of variables, including: the presence and nature of interpersonal interactions among household occupants regarding thermal comfort and/or thermostat use; presence of thermostat adjustments; and statements about thermal comfort. The results of this coding process yielded the set of variables described below. Inter-rater reliabilities ranged from 0.76 for thermal comfort to 0.88 for interaction type. Disagreements were resolved through discussion.

Interactions. An interaction was defined as having occurred when a diary response described any discussion among household occupants regarding thermal comfort. A total of three specific interaction types were identified, defined by the presence of (dis)agreement at the beginning and/or end of the interaction: (1) “agreement” was defined as having occurred when two or more household occupants agreed with respect to their thermal comfort (and subsequent course of action) at both the beginning and end of the interaction, (2) “conflict” was defined as occurring when two or more household occupants disagreed at both the beginning and end of the interaction, and (3) “compromise” was defined as occurring when two or more household occupants disagreed at the beginning of the interaction but agreed by the end of it. A set of three binary variables was created to indicate the three specific interaction types (i.e., agreement, compromise, and conflict), each of which was coded as “0” = did not occur and “1” = occurred on a given day. We also created a “nonspecific” interaction variable to reflect instances whereby participants reported information suggesting that an interaction occurred but did not provide sufficient detail to classify it into one of the three aforementioned types. The nonspecific interaction variable was coded as “0” = did not occur and “1” = occurred, for each given day. An additional variable, “any interaction”, was created to aggregate the above coding and indicates whether or not any type of interaction (including any of the three specific interaction types or a nonspecific interaction) took place on a given day; “any interaction” is coded as “1” if any interaction occurred on a given day, and “0” if none did.

Thermostat adjustments. A variable was created to reflect whether or not the participant reported that a thermostat adjustment was made in their home on a given day. There was considerable variability in the ways participants described thermostat adjustments (e.g., raise/lower the temperature vs. turn up/down the heat/air conditioning), and many participants did not report directionality at all, making it impossible to define thermostat adjustments as energy using or energy saving. Thus, our thermostat adjustment variable focuses only on whether or not an adjustment occurred on a given day (“0” = no, “1” = yes), and does not indicate direction of adjustment.

Experienced thermal discomfort. We created a set of variables reflecting thermal discomfort based on participants’ reports of household occupants identifying their thermal state relative to the ambient temperature of the home (i.e., not to outdoor temperature). For each given day, if a participant mentioned their own or anyone else in the household’s comfort, the instance was given two codes, one coding the valence of the assessment (hot, cold, or comfortable), and another coding the actor(s) involved. For actor coding, “self” or “other” codes were assigned when participants described that either they themselves or other occupants assessed the thermal environment, respectively; a “joint” code was applied in cases of a shared assessment between the participant and one or more other occupants. To code for valence, for a given day, if a participant mentioned that anyone in the household felt that the thermal environment was below preferred (i.e., using the word “cold”, “cool”, “chilly”, etc.), a “cold” code was assigned. A binary “cold” variable was created, coded as “1” = cold code present on a given day and “0” otherwise. Likewise, a “hot” code was created to describe days on which participants reported that anyone in their household felt the household temperature was above preferred (e.g., “hot”, “too warm”, etc.). A binary “hot” variable was created, with each day coded as “1” = hot code present and “0” otherwise. Participants who reported that the ambient temperature was acceptable for occupants in their household (i.e., temperature is “fine”, “comfortable”, etc.) received a “comfortable” code. A binary “comfortable” variable was created, coded as “1” = comfortable code present and “0” otherwise. We then created a binary variable to represent “household thermal discomfort”, when discomfort was experienced by any household occupant, coded as “1” if either or both of the “cold” or “hot” codes were present on a given day, and “0” otherwise.

To code for whether the participant (as opposed to another occupant) was uncomfortable on a given day, we created a “participant discomfort variable”. First, we coded every instance where a participant was involved in assessing household thermal discomfort (as indicated by the presence of the “self” actor code) as “1”. For 12 participant-days, this included multiple actor and/or valence codes; for these cases, we manually checked whether discomfort assessments were made by the participant or another occupant. These instances were manually coded “1” if the participant was associated with the “hot” or “cold” code, and “0” otherwise. This resulted in a binary “participant thermal discomfort” variable where “1” indicates that the participant experienced thermal discomfort on a given day, and “0” otherwise.

### Participants

A total of 330 individuals initially agreed to participate in the study by signing up in Remind. Of these, 131 provided no diary data and hence could not be included, leaving us with data for 199 households. Participants were then dropped from the sample for the following reasons. Households with only a single person were dropped, due to our focus on interactions (n = 7). Among eight households from which two participants from the same household enrolled, one participant was randomly dropped assigned by coin toss (n = 8). Following this, 59 participants were dropped for not answering key survey questions (namely gender, age, whether or not the thermostat was programmed, or more than one question constituting the preference for warmth, preference for cool, or bill consciousness scales). Finally, participants who provided diary data on fewer than seven days were dropped (n = 13).

Our full sample thus includes N = 112 households, who provided N = 1568 household-days of diary observations (panel sample). See Table 1 for sample characteristics.

**Table 1:** Sample characteristics (n = 112 participants) relative to Franklin County, State of Ohio, and the U.S. [41,42].

	Mean (SD) or %			
Participant Characteristic	Sample	Franklin County	Ohio	U.S.
Gender (% women)	54%	51%	51%	51%
Education > = bachelors	93%	39%^1^	27%^1^	31%^1^
Race (% white)	96%	68%	82%	77%
Ethnicity: Hispanic, Latino, or Spanish origin	3%	6%	4%	18%
Household income > $100k	79%	Median: $56,319	Median: $52,407	Median: $57,652
Median age	50	34	39	38
Political orientation (% liberal)	43%	--	--	26%^2^
**Household characteristics**				
Occupancy				
1 person	--	32%	30%	28%
2 persons	29%	32%	35%	34%
3 persons	31%	15%	15%	16%
4 or more persons	40%	20%	20%	23%
Thermostat is programmed	81%			
Thermostat adjusted at least once	75%			

^1^ Of people age 25 and over

^2^ Political orientation in Gallup was measured using a five-point scale (i.e., very conservative, conservative, moderate, liberal, very liberal). In our survey, political orientation was measured using a similar seven-point scale that included “slightly conservative/liberal” options. For comparability to the Gallup results, “% Liberal” in Table 1 does not include those in our sample who identified as “slightly liberal”. Including the “slightly liberal” option, liberals constituted 52% of the sample.

#### Dropout analyses

We use two-tailed t-tests and report Cohen’s d effect sizes to evaluate potential differences between ‘completers’ (N = 112) and ‘non-completers’ with the requisite data (N = 72) in terms of subjective need for warmth, subjective need for cool, and bill consciousness scales; a large effect size is >0.8, a medium >0.5, and a small >0.2 [43]. No significant differences between completers vs. non-completers are observed for subjective need for warmth (M_c_ = 3.69, M_n_ = 3.74, *p* = 0.84, *d* = 0.03) or cool (M_c_ = 3.75, M_n_ = 3.82, *p* = 0.78, *d* = 0.04), or mean bill consciousness (M_c_ = 5.14, M_n_ = 5.22, *p* = 0.67, *d* = 0.06).

We next use two-tailed t-tests to examine whether the ratio of adjustments, interactions, and discomfort to active days was higher for completers (N = 112) vs. non-completers (N = 72). We find no significant differences between completers and non-completers in the number of days when thermostats were adjusted relative to the number of active days (*p* = 0.51, M_c_ = 0.26, M_n_ = 0.29, *d* = 0.10), the ratio of reported discussions to active days (*p* = 0.26, M_c_ = 0.17, M_n_ = 0.14, *d* = 0.17), nor the ratio of reports of discomfort to active days (*p* = 0.38, M_c_ = 0.18, M_n_ = 0.16, *d* = 0.13).

Finally, we use a simple logit with survey completion as the dependent variable (incomplete = 1) to examine whether gender, or having a thermostat previously programmed, predicted greater likelihood of survey completion. Neither variable reached traditional levels of significance, but participants who had programmed their thermostats were marginally more likely to complete surveys (gender β = -1.06, p = 0.10; thermostat programmed β = -1.11, p = 0.08).

## Results

See Table 2 for descriptive statistics on key variables used in analyses. To test H1a and H1b, we conduct two separate two-tailed t-tests with preference for warmth and preference for cool as the dependent variables, respectively. We also report Cohen’s d effect sizes. Women report significantly stronger preference for warm thermal environments compared to men (M_w_ = 4.00, N_w_ = 60, M_m_ = 3.34, N_m_ = 52, *p* = 0.02, *d* = 0.45). A marginal difference in preference for cool between genders is observed, whereby men trend towards preferring cooler environments than women (M_f_ = 3.49, N_w_ = 60, M_m_ = 4.05, N_m_ = 52, *p* = 0.07, *d* = 0.34). We thus find support for H1a and modest partial support for H1b.

**Table 2:** Descriptive statistics on key variables (n = 112 participants).

	Total count	Mean (SD) count	Range
Provided diary response	1413	12.62 (1.95)	7–14
Any interaction^1^	243	2.17 (2.77)	0–13
Agreement	55	0.49 (1.46)	0–13
Compromise	58	0.52 (0.83)	0–4
Conflict	31	0.28 (0.57)	0–3
Thermostat adjusted	353	3.15 (3.19)	0–13
Participant discomfort	57	0.51 (0.96)	0–5
Household discomfort^2^	250	2.23 (2.31)	0–10
	Mean (SD)		
Bill consciousness		5.14 (1.29)	1–7
Preference for warmth		3.69 (1.51)	1–7
Preference for cool		3.75 (1.65)	1–7

^1^Includes nonspecific interactions.

^2^Any occupant in a given household (including participant) experienced thermal discomfort.

We test H2 using Wilcoxon rank-sum tests (given the non-normal distribution of the dependent variable). H2 is supported, with women participants reporting discomfort on significantly more days than men participants (M_w_ = 0.77, N_w_ = 60, M_m_ = 0.21, N_m_ = 52, *z* = -3.6, *p* = 0.000, *d* = 0.60).

To test H3a-c and H4a-b, we use three-stage fixed effects vector decomposition analyses [44,45]. The fixed effects specification can be advantageous relative to a random effects model due to the assumption in the latter that unobserved heterogeneity is uncorrelated with independent variables [44,46,47]. We tested the validity of this assumption using a Hausman test, and determined that our data do not meet the distributional assumptions [47]. Hence, separately for H3a-c and H4a-b, we use the following three-stage approach. In the first model stage, we estimate a logit model with fixed participant-level effects while controlling for time-varying independent variables (i.e., H3: thermal discomfort; H4: the interaction types, household discomfort). In the second stage, we estimate a pooled ordinary least squares (OLS) model to partition the fixed (participant-level) estimate from stage 1 into three separate components: the time-invariant variables of interest (H3: participant gender and age; H4: bill consciousness and thermostat programmed), and a residual component that is independent of these variables of interest, and which captures all remaining unobserved time-invariant heterogeneity [45,46]. Finally, in the third stage, we estimate a logit model similar to that in stage 1, but including all three of the partitioned estimates from the second stage. These partitioned controls allow for the possibility that unobserved differences across participants may be correlated with the time-varying variables, while allowing us to recover the coefficients on the time-invariant variables of interest. In all three stages, standard errors are clustered at the participant level.

With fixed effects models containing repeated observations from the same participant as in the present case, inclusion of participants without variation in the dependent variable is not preferred as this can artificially inflate model accuracy [47]. Thus, H3a-c and H4a-b models included only participants who had variation in the dependent variable over the diary period [47]. That is, if a participant always or never reported a given interaction type, or always or never adjusted their thermostat (n = 30), they were dropped from the H3a-c or H4a-b analyses, respectively. Data for the H3a-c and H4a-b models are not balanced; as long as households have at least seven days of diary data, they are included regardless of the number of days between 7–14 on which they provided data.

In the models testing H3a-c, dependent variables are the interaction types (i.e., any interaction, agreement, compromise, conflict), each modelled separately in models I-IV. Gender is the key independent variable, with age, household discomfort, and the residual fixed effect included as covariates. We examine a dichotomous outcome: whether or not an interaction of a given type occurred on a given day. Output from the third model stage is presented in Table 3 below, and interim results from the first and second stages are shown in S2 Appendix. We find that household discomfort on a given day significantly and positively predicts the likelihood of any interaction that day, supporting H3a. In other words, any household occupant experiencing thermal discomfort on a given day significantly elevates the likelihood of a negotiation with other occupants ensuing on that day. This effect is seen across each specific interaction type, and is strongest for conflict. Examining the influence of gender on interaction types, results support H3b, with modest partial support for H3c. A significant negative coefficient for gender is found in the agreement and compromise models, and a marginally significant positive coefficient in the conflict model. These results indicate that compared to men participants, women participants are less likely to report engaging in agreements and compromises, and are marginally more likely to report engaging in conflicts on a given day. Participant age is not a significant predictor of interactions.

**Table 3:** Impacts of thermal discomfort and participant gender on likelihood of each interaction type.

	Model I	Model II	Model III	Model IV
	Any interaction	Agreement	Compromise	Conflict
Household discomfort	3.30*** (0.48)	2.46** (0.77)	4.07*** (0.55)	4.39*** (0.70)
Participant gender	-0.34 (0.33)	-1.44*** (0.41)	-0.52* (0.23)	0.28^+^ (0.14)
Participant age	-0.01 (0.01)	-0.02 (0.02)	0.01 (0.01)	0.01 (0.01)
Residual fixed effects	3.80* (1.74)	4.11 (3.00)	6.39*** (1.28)	6.67*** (1.55)
Wald χ^2^_(df = 4)_	100.79***	30.20***	77.57***	80.43***
Pseudo R^2^	0.19	0.13	0.23	0.23
n	928	364	510	331

Standard errors clustered at participant level; in parentheses.^.^

^+^ *p* < 0.10

* *p* < 0.05

** *p* < 0.01

*** *p* < 0.001.

In testing H4a and H4b, the dependent variable is whether or not the thermostat was adjusted on a given day. Separate models (V-VIII) for each interaction type as a key independent variable are run. Output from the third model stage is presented in Table 4 below, and interim results from the first and second stages are detailed in S3 Appendix. Across all models, having the thermostat programmed has no effect on whether thermostat adjustments are made on a given day. However, whether an interaction occurs on a given day does influence likelihood of thermostat adjustments being made on that day. Furthermore, the type of interaction matters, but in the opposite directions of our H4a and H4b predictions. Specifically, participants are more likely to report thermostat adjustments being made on days on which they engaged in an agreement or compromise. However, we see the opposite pattern for conflicts: participants are less likely to report thermostat adjustments on days on which they engaged in conflicts. For days on which any household occupant experienced discomfort, thermostat adjustments are also more likely. Bill consciousness is not a significant predictor of thermostat adjustments.

**Table 4:** Impacts of interactions on likelihood of thermostat adjustments (N = 1040 observations from N = 82 participants).

	Model V	Model VI	Model VII	Model VIII
Any interaction	0.79** (0.30)			
Agreement interaction		0.95* (0.48)		
Compromise interaction			0.85* (0.41)	
Conflict interaction				-1.36** (0.51)
Household discomfort	1.47*** (0.28)	2.21*** (0.45)	1.63*** (0.37)	2.81*** (0.53)
Thermostat programmed	-0.47 (0.43)	-0.41 (0.43)	-0.45 (0.43)	-0.41 (0.42)
Bill consciousness	-0.15 (0.09)	-0.15^+^ (0.09)	-0.12 (0.09)	-0.14 (0.09)
Residual fixed effects	2.51 (1.70)	4.45* (2.16)	2.03 (1.80)	6.38** (2.44)
Wald χ^2^_(df = 5)_	83.88***	84.52***	84.72***	86.56***
Pseudo R^2^	0.07	0.07	0.07	0.07

Standard errors clustered at participant level; in parentheses.

^+^ *p* < 0.10

* *p* < 0.05

** *p* < 0.01

*** *p* < 0.001.

## Discussion

Heating and cooling consume a large share of residential energy use worldwide [2], and smart and programmable thermostats have potential for home energy savings [4] [5]. However, people often do not use these devices in ways that fully exploit their energy efficiency potential [6,7]. In other words, such savings also hinge on human behaviour [8,10], necessitating consideration of household behavioral dynamics that may contribute to this phenomenon. The present study is the first to our knowledge to examine types of intra-household interactions around thermal comfort, and their impacts on home energy use behavior. We identify three types of interactions: conflicts, compromises, and agreements. Building on emerging research that discussing energy use can be linked to energy efficiency decisions (Southwell & Murphy, 2014), our results indicate that having an interaction on a given day impacts likelihood of adjusting the thermostat on that day. Further, the type of interaction in which occupants engage on a particular day can be associated with either elevated or reduced likelihood of a thermostat adjustment that day; merely examining the presence of “any interaction”, without considering its nature, obscures such directional effects. Hence, it is critical to account for the nature of interactions in understanding their influence on energy-using activities. These findings represent a modest, early step in a broader line of inquiry that is sensitive to the multiply determined nature of household energy use behavior.

### The role of gender in thermal comfort interactions

As has been highlighted previously in relation to smart energy monitors [28], understanding how household occupants negotiate decisions around thermostats offers a peek into household decision-making dynamics, including a unique context for studying the role of gender in negotiations. Replicating prior findings, we find significant gender variation in both subjective thermal comfort preferences as well as experience of thermal discomfort. Specifically, women report greater subjective need for warmth, and experience thermal discomfort more often than men [11,18,19,22]. This latter finding implies a gender bias in house-wide thermal comfort settings, whereby home thermal environments do not cater to women’s preferences.

When it comes to negotiating thermal comfort preferences with other occupants, men are more likely to report agreements and compromises as outcomes, whereas women are marginally more likely to report conflicts. One interpretation of these findings is that when men are negotiating for their thermal comfort needs, their negotiation partners may be more likely to “give in”, whereas women may not be met with this same outcome. Prior work also shows that women suffer reduced cognitive performance in colder temperatures, whereas men perform better in such environments [20]. It is therefore also possible that men have a comparative advantage in negotiations that occur in cooler environments. Future work should examine these possibilities, which our data cannot address.

It is important to note that our data also do not indicate who initiated thermal comfort interactions. In line with previous work, it is possible that women are less likely to initiate such negotiations [34,48]. Consistent with the woman gender role characteristics of being accommodating and relationship-oriented [36,37], women may instead take individual-level action to improve their thermal comfort (e.g., putting on a sweater), or simply endure discomfort, deferring to the preferences of others [38]. Our data cannot speak to these possibilities; future work should examine them.

### The influence of interactions on energy use behavior

How do thermal comfort interactions influence thermostat adjustments? Having an interaction that ends in agreement (i.e., agreement and compromise) on a particular day is associated with higher likelihood of adjusting the thermostat that same day, suggesting that adjusting the thermostat may serve as a means to appease the household. On the other hand, conflict is associated with lower likelihood of thermostat adjustments within the same day. In light of our finding that women report thermal discomfort more frequently, this suggests that their desired thermal comfort outcomes are more often not achieved relative to men. Prior work finds that women are less likely to negotiate in situations in which they perceive lowered negotiation outcomes or psychological power [48]. Additionally, individuals who possess or perceive greater power in a given situation are more likely to act in ways consistent with desired end-states [49]. Following this line of reasoning, occupants who have or perceive greater power in decision-making over household equipment may be more likely to “take control” of decisions to achieve their desired thermal comfort state. Gender differences in occupants’ interactions with home energy technologies, such as smart energy monitors, have been observed in prior work, suggesting that men are often responsible for home equipment related to heating and cooling [28,29]. Linking these findings to the adaptive thermal comfort model [14,16], if women have lower levels of perceived control over home heating and cooling in their homes, this may contribute to their more frequent dissatisfaction with the thermal environment. Indeed, prior work finds that women perceive that they have less control over thermal settings at home, feel uncomfortable more often, and adjust the thermostat less often relative to men [11].

In sum, women experience more frequent dissatisfaction with thermal comfort, suggesting a status quo gender bias in the household thermal environment. Further, when engaged in negotiations over thermal comfort, the outcome reported by women trends toward conflict, versus agreement or compromise being significantly more likely for men—interactions associated with thermostat adjustments on a given day—representing additional dissatisfaction for women on top of thermal discomfort.

### Implications for residential energy efficiency

For thermostats to achieve maximum energy efficiency, energy saving programs should be set and occupants should let them run. However, in this study we observed that whether or not someone programs their thermostat does not influence the likelihood of making manual adjustments on a given day. In other words, even when people set programs on thermostats, they don’t always let these programs run, which can interfere with energy efficiency goals. These findings contribute to the existing body of evidence suggesting that despite the promises of energy efficient technology, the technology alone is not sufficient to achieve energy savings [10,50]. Rather, the ways in which people interact with such technologies–and, as found in this study, the ways in which people interact with one another–modulate the devices’ potential for energy savings. Given the gender differences in interaction types, perhaps in the future, building thermal management systems can act as mediators of interactions between occupants. For instance, multiple occupants could individually input into a home system (e.g., via a mobile phone app or website) their preferred temperatures and whether or not they feel comfortable in real time. The system could then negotiate these multiple dynamic preferences in real-time, aiming to optimize overall comfort and minimize energy use/cost. Prior simulations and small (n<10) in-situ demonstrations of such a system suggest it may be feasible [26,51], but future work is needed to field-test such a solution and examine potential gender differences in participation in such a system.

### Limitations and future directions

This work should be considered in light of its limitations. First, many participants did not continue in the study following initial recruitment. Additionally, our sample is older, more educated, more liberal, has higher income levels, and is less racially diverse than the general population. We also only had participants who identified as a woman or a man, limiting the gender identities represented by our findings. Hence, results may not generalize to other settings or populations.

Additionally, our sample consisted of individuals reporting on behalf of their households. It is possible that there are gender differences in how a conversation is perceived and/or reported (e.g., if women are more bothered by a disagreement, they might be more likely to report it, and/or report it as a conflict vs. a compromise). Related, we did not gather data on conversation initiation nor analyse specific content of conversations. To address these limitations, future work should attempt to recruit multiple members of a given household, and gather data on the genders of all parties involved in a negotiation, who initiates conversations, as well as analyze conversation content.

Another limitation of the present study is that data collection occurred in one season, Autumn, during which outdoor temperatures were dropping in central Ohio. This study should be repeated in a season during which outdoor temperatures are rising, or stable, to determine the extent to which results generalize under different outdoor weather conditions. Future studies should also account for time of day of reports, given diurnal fluctuations in outdoor temperatures. Finally, data are self-report, and due to the open-ended nature of diary responses, we were unable to determine the direction of thermostat adjustments and whether they were energy-using or energy-saving. Hence, the results of the study do not have clear implications for intervention. Future work should provide for capture of these details as well as observed behavioral measures.

## Supporting information

S1 Appendix Key survey measures. (DOCX)

S2 Appendix First and second stage results for research question 3. (DOCX)

S3 Appendix First and second stage results for research question 4. (DOCX)

## References

[1] EIA. 2015 Residential Energy Consumption Survey: Energy Consumption and Expenditures Tables. 2018.

[2] Ürge-Vorsatz D, Cabeza LF, Serrano S, Barreneche C, Petrichenko K. Heating and cooling energy trends and drivers in buildings. Renew Sustain Energy Rev. 2015;41: 85–98. 10.1016/j.rser.2014.08.039

[3] Nelson LW, MacArthur JW. Energy Savings Through Thermostat Setbacks. ASHRAE Trans. 1978;83: 319–333.

[4] Sanchez M. Savings estimates for the United States Environmental Protection Agency’s ENERGY STAR voluntary product labeling program. Also Appear Energy Policy. 2008 Available: https://cloudfront.escholarship.org/dist/prd/content/qt9c59w1gq/qt9c59w1gq.pdf

[5] Manning MM, Swinton MC, Szadkowski F, Gusdorf J, Ruest K. The Effects of thermostat setting on seasonal energy consumption at the CCHT Twin House Facility Maison expérimentale-Projet avec l’industrie et le Gouvernement fédéral View project Exterior Insulation Basement Systems (EIBS) View project. 2007. Available: http://irc.nrc-cnrc.gc.ca

[6] Meier A, Aragon C, Hurwitz B, Mujumdar D, Peffer T, Perry D, et al How People Actually Use Thermostats. Berkeley, CA; 2010.

[7] Pritoni M, Meier AK, Aragon C, Perry D, Peffer T. Energy efficiency and the misuse of programmable thermostats: The effectiveness of crowdsourcing for understanding household behavior. Energy Res Soc Sci. 2015;8: 190–197. 10.1016/j.erss.2015.06.002

[8] Peffer T, Pritoni M, Meier A, Aragon C, Perry D. How people use thermostats in homes: A review. Build Environ. 2011;46: 2529–2541. 10.1016/j.buildenv.2011.06.002

[9] Peffer T, Perry D, Pritoni M, Aragon C, Meier A. Facilitating energy savings with programmable thermostats: Evaluation and guidelines for the thermostat user interface. Ergonomics. 2013;56: 463–479. 10.1080/00140139.2012.718370 23005033

[10] Malinick T, Wilairat N, Holmes J, Perry L, Ware W. Destined to Disappoint: Programmable Thermostat Savings are Only as Good as the Assumptions about Their Operating Characteristics ENERGY STAR and Programmable Thermostats. 2012.

[11] Karjalainen S. Gender differences in thermal comfort and use of thermostats in everyday thermal environments. Build Environ. 2007;42: 1594–1603. 10.1016/j.buildenv.2006.01.009

[12] Walker IS, Meier AK. Residential Thermostats: Comfort Controls in California Homes. Berkeley, CA; 2008.

[13] Brager GS, de Dear R. Thermal Adaptation in the Built Environment: a Literature Review. Energy Build. 1998;27: 83–96. Available: https://escholarship.org/content/qt5ts1r442/qt5ts1r442.pdf

[14] de Dear R, Brager GS. Developing an adaptive model of thermal comfort and preference. 1998 Available: https://escholarship.org/uc/item/4qq2p9c6#author

[15] de Dear RJ, Akimoto T, Arens EA, Brager G, Candido C, Cheong KWD, et al Progress in thermal comfort research over the last twenty years. Indoor Air. 2013;23: 442–461. 10.1111/ina.12046 23590514

[16] Paciuk MT. The role of personal control of the environment in thermal comfort and satisfaction at the workplace. University of Wisconsin at Milwaukee 1989 Available: https://elibrary.ru/item.asp?id=5898973

[17] Brager G, Zhang H, Arens E. Evolving opportunities for providing thermal comfort. Build Res Inf. 2015;43: 274–287. 10.1080/09613218.2015.993536

[18] Eon C, Morrison GM, Byrne J. Unraveling everyday heating practices in residential homes. Energy Procedia. 2017;121: 198–205. 10.1016/j.egypro.2017.08.018

[19] Becker LJ, Seligman C, Fazio RH, Darley JM. Relating attitudes to residential energy use. Environ Behav. 1981;13: 590–609.

[20] Chang TY, Kajackaite A. Battle for the thermostat: Gender and the effect of temperature on cognitive performance. Capraro V, editor. PLoS One. 2019;14: e0216362 10.1371/journal.pone.0216362 31116745 PMC6530830

[21] Schweiker M, Huebner GM, Kingma BRM, Kramer R, Pallubinsky H. Drivers of diversity in human thermal perception–A review for holistic comfort models. Temperature. 2018;5: 308–342. 10.1080/23328940.2018.1534490 30574525 PMC6298492

[22] Chen C fei, Xu X, Day JK. Thermal comfort or money saving? Exploring intentions to conserve energy among low-income households in the United States. Energy Res Soc Sci. 2017;26: 61–71. 10.1016/j.erss.2017.01.009

[23] International Energy Agency. Transition to Sustainable Buildings: Strategies and Opportunities to 2050. 2013.

[24] United Nations Department of Economic and Social Affairs Population Division. Household Size and Composition Around the World 2017 –Data Booklet (ST/ESA/ SER.A/405). 2017.

[25] de Dear R, Brager GS. The adaptive model of thermal comfort and energy conservation in the built environment. Int J Biometeorol. 2001;45: 100–108. 10.1007/s004840100093 11513046

[26] Gupta SK, Kar K, Mishra S, Wen JT. Collaborative Energy and Thermal Comfort Management Through Distributed Consensus Algorithms. IEEE Trans Autom Sci Eng. 2015;12: 1285–1296. 10.1109/TASE.2015.2468730

[27] Southwell BG, Murphy J. Weatherization behavior and social context: The influences of factual knowledge and social interaction. Energy Res Soc Sci. 2014;2: 59–65. 10.1016/j.erss.2014.03.019

[28] Hargreaves T, Nye M, Burgess J. Making energy visible: A qualitative field study of how householders interact with feedback from smart energy monitors. Energy Policy. 2010;38: 6111–6119. 10.1016/J.ENPOL.2010.05.068

[29] Carlsson-Kanyama A, Lindén A-L. Energy efficiency in residences—Challenges for women and men in the North. Energy Policy. 2007;35: 2163–2172. 10.1016/j.enpol.2006.06.018

[30] Abrahamse W, Steg L, Vlek C, Rothengatter T. A review of intervention studies aimed at household energy conservation. J Environ Psychol. 2005;25: 273–291. 10.1016/j.jenvp.2005.08.002

[31] Delmas MA, Fischlein M, Asensio OI. Information strategies and energy conservation behavior: A meta-analysis of experimental studies from 1975 to 2012. Energy Policy. 2013;61: 729–739. 10.1016/j.enpol.2013.05.109

[32] Hwang Y, Southwell BG. Can a Personality Trait Predict Talk About Science? Sci Commun. 2007;29: 198–216. 10.1177/1075547007308599

[33] Mazei J, Hüffmeier J, Freund PA, Stuhlmacher AF, Bilke L, Hertel G. A meta-analysis on gender differences in negotiation outcomes and their moderators. Psychol Bull. 2015;141: 85–104. 10.1037/a0038184 25420223

[34] Small DA, Gelfand M, Babcock L, Gettman H. Who Goes to the Bargaining Table? The Influence of Gender and Framing on the Initiation of Negotiation. J Pers Soc Psychol. 2007;93: 600–613. 10.1037/0022-3514.93.4.600 17892334

[35] Babcock L, Laschever S. Women don’t ask: negotiation and the gender divide. Princeton University Press; 2003.

[36] Eagly AH, Wood W. The origins of sex differences in human behavior: Evolved dispositions versus social roles. Am Psychol. 1999;54: 408–423.

[37] Eagly AH, Karau SJ. Role congruity theory of prejudice toward female leaders. Psychol Rev. 2002;109: 573–598. 10.1037/0033-295x.109.3.573 12088246

[38] Holt JL, DeVore CJ. Culture, gender, organizational role, and styles of conflict resolution: A meta-analysis. Int J Intercult Relations. 2005;29: 165–196. 10.1016/j.ijintrel.2005.06.002

[39] Kray LJ, Thompson L, Galinsky A. Battle of the Sexes: Gender Stereotype Confirmation and Reactance in Negotiations. J Pers Soc Psychol. 2001;80: 942–958. 10.1037/0022-3514.80.6.942 11414376

[40] Saldana J. Chapter 1. An Introduction to Codes and Coding. Coding Man Qual Res. 2015; 1–31.

[41] United States Census Bureau. 2013–2017 American Community Survey 5-year Estimates. 2017.

[42] Gallup. Conservative Lead in U.S. Ideology Is Down to Single Digits. 2018.

[43] Cohen J. Statistical Power Analysis for the Behavioral Sciences. 2nd Editio. Hillsdale, NJ: Lawrence Earlbaum Associates; 1988.

[44] Plümper T, Troeger VE. Fixed-Effects Vector Decomposition: Properties, Reliability, and Instruments. Polit Anal. 2011;19: 147–164. 10.1093/pan/mpr008

[45] Plümper T, Troeger VE. Efficient Estimation of Time-Invariant and Rarely Changing Variables in Finite Sample Panel Analyses with Unit Fixed Effects. Polit Anal. 2007;15: 124–139. 10.1093/pan/mpm002

[46] Abowd JM, Kramarz F, Margolis DN. High Wage Workers and High Wage Firms. Econometrica. 1999;67: 251–333. 10.1111/1468-0262.00020

[47] Wooldridge JM. Econometric analysis of cross section and panel data. MIT Press; 2002.

[48] Leier CR, Alberts J, Miller K, Hinshaw A. Why Don’t Women Ask? A Mixed Method Analysis of Gender and the Propensity to Initiate a Negation. Arizona State University 2015.

[49] Galinsky AD, Gruenfeld DH, Magee JC. From Power to Action. J Pers Soc Psychol. 2003;85: 453–466. 10.1037/0022-3514.85.3.453 14498782

[50] Sintov ND, Schultz PW. Unlocking the potential of smart grid technologies with behavioral science. Front Psychol. 2015;6 10.3389/fpsyg.2015.00410 25914666 PMC4391202

[51] Gupta SK, Atkinson S, O’Boyle I, Drogo J, Kar K, Mishra S, et al BEES: Real-time occupant feedback and environmental learning framework for collaborative thermal management in multi-zone, multi-occupant buildings. Energy Build. 2016;125: 142–152. 10.1016/J.ENBUILD.2016.04.084

