# Supplementary material for: Thermostat wars? The roles of gender and thermal comfort negotiations in household energy use behavior
Source: PLoS One. 2019 Nov 13;14(11):e0224198. doi: 10.1371/journal.pone.0224198 (PMC6853289; doi:10.1371/journal.pone.0224198)
Supplement: S1 Appendix — (DOCX) [file pone.0224198.s001.docx]

**Supporting Information 1: Key Survey Measures**

**Bill consciousness (from Chen et al., 2017)**

For the following statements, please tell us how much you disagree or agree with each by selecting the corresponding bubble.

(Responses provided on a scale from Strongly disagree (1) – Strongly agree (7))

I pay attention to energy saving tips to reduce my energy bills

I am motivated to keep my monthly energy costs under a reasonable amount

I keep track of my monthly energy bills

**Thermal comfort preferences (from Chen et al., 2017)**

For the following statements, please tell us how much you disagree or agree with each by selecting the corresponding bubble.

(Responses provided on a scale from Strongly disagree (1) – Strongly agree (7))

I find I cannot relax or work well unless the house is air conditioned in the summer

I have trouble falling asleep at night without an air conditioner on

While others might turn off their air conditioners in the summer, my own need for being cool is high

It is just too uncomfortable when my home's indoor temperature feels cold in winter months

While others might tolerate lowering their thermostat settings in the winter, my own need for being warm is high
