# Supplementary material for: Thermostat wars? The roles of gender and thermal comfort negotiations in household energy use behavior
Source: PLoS One. 2019 Nov 13;14(11):e0224198. doi: 10.1371/journal.pone.0224198 (PMC6853289; doi:10.1371/journal.pone.0224198)
Supplement: S2 Appendix — (DOCX) [file pone.0224198.s002.docx]

**Supporting Information 2: First and second stage results for research question 3**

To investigate our third research question, we use three-stage fixed effects vector decomposition analyses (44,45). In the first model stage, we estimate a logit model with fixed participant-level effects while controlling for our time-varying independent variable: household discomfort.

**Table S1: First Stage Model for H3a-c: Logit Model with Interaction Types as the Dependent Variables.**

|  | Model I | Model II | Model III | Model IV |
| --- | --- | --- | --- | --- |
|  | Any interaction | Agreement | Compromise | Conflict |
| Household discomfort | 2.88*** (0.39) | 1.95*** (0.44) | 2.88*** (0.44) | 2.83*** (0.61) |
| Wald χ^2^_(df=1)_ | 54.94*** | 20.04*** | 43.18*** | 21.69*** |
| Pseudo R^2^ | 0.27 | 0.13 | 0.27 | 0.26 |
| N | 928 | 365 | 510 | 331 |

Standard errors clustered at participant level; in parentheses.^. *^ *p* < 0.05, ^**^ *p* < 0.01, ^***^ *p* < 0.001.

In the second stage, we estimate a pooled ordinary least squares (OLS) model to partition the fixed (participant-level) estimate from stage 1 into three separate components: participant gender and age, and a residual component that is independent of gender and age, and which captures all remaining unobserved time-invariant heterogeneity (45,47).

**Table S2: Second Stage Model for H3a-c: Pooled OLS Model with the Dependent Variable Being the ‘Fixed Effect’ Term from the First Stage.**

|  | Model I | Model II | Model III | Model IV |
| --- | --- | --- | --- | --- |
|  | Any interaction | Agreement | Compromise | Conflict |
| Participant gender | -0.03 (0.02) | -0.04 (0.03) | -0.02 (0.03) | -0.03 (0.03) |
| Participant age | 0.00 (0.00) | 0.00 (0.00) | 0.00 (0.00) | 0.00** (0.00) |
| F  (df) | (2, 71) | (2, 27) | (2, 38) | (2, 24) |
| F statistic | 2.18 | 2.12 | 1.44 | 6.11 |
| R^2^ | 0.02 | 0.04 | 0.02 | 0.04 |
| N | 928 | 364 | 510 | 331 |

Standard errors clustered at participant level; in parentheses.^. *^ *p* < 0.05, ^**^ *p* < 0.01, ^***^ *p* < 0.001.

Finally, in the third stage, presented in Table 3 of the manuscript, we estimate a logit model similar to that in stage 1, but including all three partitioned estimates from the second stage. These partitioned controls allow for the possibility that unobserved differences across participants may be correlated with the time-varying variables, while allowing us to recover the coefficients on the time-invariant variables of interest, gender and age. In all three stages, standard errors are clustered at the participant level.
