# Supplementary material for: Thermostat wars? The roles of gender and thermal comfort negotiations in household energy use behavior
Source: PLoS One. 2019 Nov 13;14(11):e0224198. doi: 10.1371/journal.pone.0224198 (PMC6853289; doi:10.1371/journal.pone.0224198)
Supplement: S3 Appendix — (DOCX) [file pone.0224198.s003.docx]

**Supporting Information 3: First and second stage results for research question 4**

To examine our fourth research question, we use three-stage fixed effects vector decomposition analyses (44,45). In the first model stage, we estimate a logit model with fixed participant-level effects while controlling for our time-varying independent variables: household discomfort and each of the interaction types.

**Table S3: First Stage Model for H4a-b: Logit Model with Thermostat Adjustment on a Given Day as the Dependent Variable (N= 1040 observations from N = 82 participants).**

|  | Model V | Model VI | Model VII | Model VIII |
| --- | --- | --- | --- | --- |
| Any interaction | 1.10*** (0.30) |  |  |  |
| Agreement interaction |  | 1.18** (0.39) |  |  |
| Compromise interaction |  |  | 1.22** (0.43) |  |
| Conflict interaction |  |  |  | -1.05 (0.66) |
| Household discomfort | 1.15*** (0.22) | 1.55*** (0.20) | 1.45*** (0.21) | 1.74*** (0.22) |
| Wald χ^2^_(df=2)_ | 72.05*** | 69.71*** | 68.88*** | 63.73*** |
| Pseudo R^2^ | 0.11 | 0.09 | 0.10 | 0.09 |

Standard errors clustered at participant level; in parentheses.^. *^ *p* < 0.05, ^**^ *p* < 0.01, ^***^ *p* < 0.001.

In the second stage, we estimate a pooled ordinary least squares (OLS) model to partition the fixed (participant-level) estimate from stage 1 into three separate components: bill consciousness and whether or not the thermostat was programmed, and a residual component that is independent of bill consciousness and thermostat programming, and which captures all remaining unobserved time-invariant heterogeneity (45,47).

**Table S4: Second Stage Model for H4a-b: Pooled OLS Model with the Dependent Variable Being the ‘Fixed Effect’ Term from the First Stage (N= 1040 observations from N = 82 participants).**

|  | Model V | Model VI | Model VII | Model VIII |
| --- | --- | --- | --- | --- |
| Thermostat programmed | -0.00 (0.02) | -0.01 (0.03) | -0.01 (0.02) | -0.01 (0.02) |
| Bill consciousness | -0.01* (0.01) | -0.01 (0.01) | -0.01 (0.01) | -0.01 (0.01) |
| F (2, 81) | 2.32 | 1.44 | 0.92 | 0.73 |
| R^2^ | 0.03 | 0.02 | 0.01 | 0.01 |

Standard errors clustered at participant level; in parentheses.^. *^ *p* < 0.05, ^**^ *p* < 0.01, ^***^ *p* < 0.001.

Finally, in the third stage, presented in Table 4 of the manuscript, we estimate a logit model similar to that in stage 1, but including all three partitioned estimates from the second stage. These partitioned controls allow for the possibility that unobserved differences across participants may be correlated with the time-varying variables, while allowing us to recover the coefficients on the time-invariant variables of interest, bill consciousness and whether or not the thermostat was programmed. In all three stages, standard errors are clustered at the participant level.
